# Eighty-Year-Old Male With Normal Pressure Hydrocephalus Presenting With Dementia and Parkinsonism Treated Using Electroencephalography (EEG)-Guided H4 Coil Protocol: A Case Report

**DOI:** 10.7759/cureus.89177

**Published:** 2025-07-31

**Authors:** Aswin Kumar Mudunuru, Kartik Valipay, Arifa Rahman, Sailaja Bomma, Lalitha V Jahnavi, M. S. Reddy

**Affiliations:** 1 Non-Invasive Brain Stimulation, Asha Neuromodulation Clinics, Hyderabad, IND; 2 Psychiatry, Asha Neuromodulation Clinics, Hyderabad, IND; 3 Neurology, Asha Neuromodulation Clinics, Hyderabad, IND; 4 Psychology, Asha Neuromodulation Clinics, Hyderabad, IND; 5 Psychiatry, Asha Hospital, Hyderabad, IND

**Keywords:** deep tms, eeg, h4 coil, idiopathic nph, parkinson's disease dementia

## Abstract

Idiopathic Normal Pressure Hydrocephalus (iNPH) is a rare syndrome of apractic or ataxic gait, mild to moderate dementia, and urinary incontinence. The cerebrospinal fluid tap test is widely used in the diagnosis of NPH. Using deep Transcranial Magnetic Simulation (DTMS) with H coils, we developed a novel treatment method that incorporates electroencephalography (EEG)-based customization of the treatment protocol. An 80-year-old male presented with clinical signs of slowness of gait and activity, rigidity, and urinary incontinence, which progressed to bradykinesia, magnetic, short, and shuffling gait. He later developed altered behavior, memory loss, and cognitive deficit. Administration of 17 DTMS sessions with an H4 coil provided transient relief from a few symptoms for approximately two months. Fast Fourier transform analysis of the frontal EEG was used to find the peak activity and customize the treatment protocol. Eleven DTMS sessions with optimized protocol gave a sustainable symptomatic relief as well as improved functionality, as estimated on 10-month follow-up.

## Introduction

Idiopathic Normal Pressure Hydrocephalus (iNPH) is a rare syndrome characterized by apractic or ataxic gait, mild to moderate dementia, and urinary incontinence, presumed to be caused by obstruction to normal flow of cerebrospinal fluid (CSF) and delayed absorption into the venous system, resulting in enlarged ventricles with relatively less increase in CSF pressure [1,2]. Both sexes are affected by the disease [3]. Characteristic imaging findings include communicating hydrocephalus with a patent aqueduct of Sylvius, without cortical or hippocampal atrophy, usually. Although there is no specific diagnostic method, the CSF tap test (CTT) is widely used in the diagnosis of iNPH [4]. The surgical treatment for iNPH involving implantation of a ventriculoperitoneal shunt (VPS) results in a favorable outcome in approximately 30%-50% of patients [5-7]. Subdural hematoma (SDH) and infection are common surgical complications [8]. A transient improvement in gait and cognitive function has also been observed following lumbar puncture (LP) with removal of >30 mL of CSF [9]. However, no treatment consistently yields predictable results across all patients, highlighting the importance of exploring alternative approaches. The non-invasive brain stimulation technique of transcranial magnetic stimulation (TMS) has shown value in diagnostic evaluation of iNPH, including as a predictor of shunt response [10]. However, it has not been used as a treatment. In this case report, we investigate the use of an optimized form of the stimulation technology known as deep TMS (DTMS) for the treatment of iNPH using EEG-based customization. This customization involves replacing the treatment frequency with extracted peak frequencies from the Fast Fourier Transform (FFT) graphs, as reported previously [11].

## Case presentation

Patient RK, a 77-year-old male, had a head injury due to an accidental fall in April 2020. CT scan showed SDH of approximately 4 mm thickness in the right fronto-parieto-temporal convexity. He developed slowness of gait and activity, difficulty in getting up from sitting posture, and urinary incontinence, which worsened over time. Five months later, a repeat CT revealed diffuse cerebral atrophy with small vessel ischemia, with no evidence of right-sided SDH. Over the next few months, symptoms worsened with the development of bradykinesia, magnetic, short, and shuffling gait, rigidity on the left side, and significant postural instability. In February 2021, there was decreased interest in activity, altered behavior, memory loss, and cognitive deficit. The diagnosis of atypical Parkinsonism was made at this time. Movement Disorder Society Unified Parkinson’s Disease Rating Scale (MDS-UPDRSIII) motor scoring was 80/132 [12]. The patient was started on Syndopa 125 mg once a day. After five months, as the symptoms were not getting controlled, the possibility of iNPH was suspected, and a CSF tap was done. Gait improved within 24 hours after the first CSF tap. Two months later, the rigidity and behavioral changes reappeared with additional findings such as backward falls during attempted walking, proximal muscle weakness, proprioceptive anesthesia, reduced arm swing, memory loss, and inability to adjust to newer environments. The patient underwent periodical CSF taps about six to eight months apart, only to witness transient improvement in all the symptoms. The family members consulted our Neuromodulation Clinic during January 2024 (aged 81 years) for the complaints of depressed mood, memory loss, and cognitive deficit. During the consultation, the previous diagnosis of iNPH was uncovered by our psychiatrist.

Examination findings

At presentation, the patient was on Syndopa 125 mg and other conventional medications with standard daily dosages for hypertension (Telmisartan 40 mg), hypercholesterolemia (Atorvastatin 20 mg), diabetes mellitus (Glimepiride and Metformin 1+500 mg), and hypothyroidism (Levothyroxine 50 μg). There was no significant finding in all other routine lab tests. The Mental Status Examination revealed tangential speech, normal eye contact, flat affect, depressed mood, distracted attention, and no hallucinations, suicidality, or delusions. He had mild depression recorded on the Hamilton Depression Rating Scale-21 (HDRS-21) as 10 points [13]. The Mini-Mental State Examination (MMSE) score was 14, and the Montreal Cognitive Assessment (MoCA) score was 12, indicating moderate cognitive impairment [14,15]. Neurological examination revealed 4/5 to 5/5 power in major muscle groups in all four limbs, marked rigidity on the left side of the body, proprioceptive anesthesia, micrographia, step length 0.4 feet (10/25), and stride length 0.8 feet (10/12.5) (measured as distance in feet/no. of steps or strides). MDS-UPDRSIII Motor Scoring was 94/132, indicating severe impairment.

First treatment plan

All the drugs were maintained status quo during DTMS treatment. Screening for electromagnetic field compatibility was done before DTMS. H4 coil was used to apply BrainsWay’s evidence-based protocol for the treatment of Parkinson’s disease [16,17]. In January 2024, 17 consecutive sessions of DTMS were given once daily, each lasting for about 30 minutes. Each session consisted of one low-frequency stimulation (LFS) of 1 Hz over the bilateral motor cortex (M1) given at 90% of resting motor threshold (RMT) with up to 900-1,400 pulses. This was followed by a high-frequency stimulation (HFS) of 10 Hz over the bilateral prefrontal cortex (PFC) given at 100% of RMT up to 800-1,200 pulses. Following this, six more maintenance sessions were given on alternate days in February 2024. The patient gained good control of urinary incontinence and a partial reduction in the rigidity. He was able to walk with support, especially after rising from sleep or in the early hours of the day. Step length was 0.5 feet (10/20) and stride length was 1 foot (10/10). However, there was no noticeable improvement in cognitive abilities and memory. In April 2024, the patient exhibited a minor worsening of his symptoms and was taken for CSF tapping. The neurologist noticed that the CSF pressure and volume were lower than before, and the immediate improvement, which was usually seen after the tapping, did not appear this time. He was brought back to the Neuromodulation Clinic during the last week of April to repeat the DTMS treatment.

**Figure 1:**
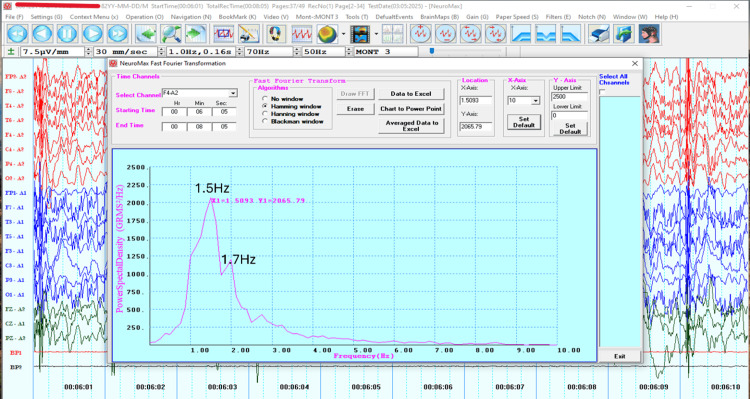
Fast Fourier Transform (FFT) graph from the F4-A2 electroencephalography (EEG) montage. The peak frequencies at 1.5 and 1.7 Hz are seen in the FFT graph of the F4-A2 montage.

**Figure 2:**
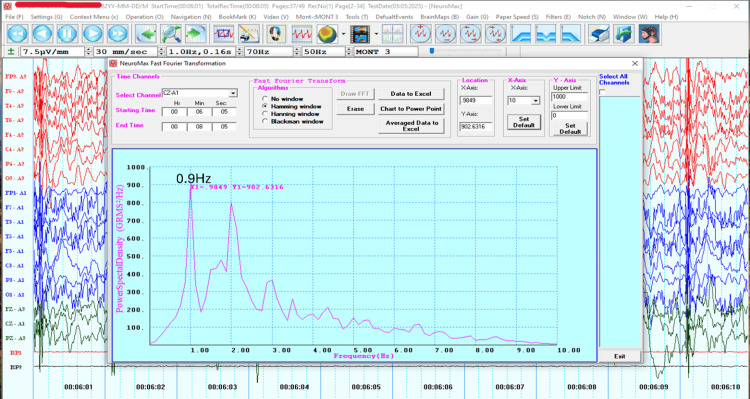
Fast Fourier Transform (FFT) graph from the Cz-A1 electroencephalography (EEG) montage. The peak frequencies at 0.9 Hz are seen in the FFT graph of the Cz-A1 montage.

Due to the limited improvement achieved thus far with DTMS treatment, it was decided to optimize the protocol by customizing it to match the patient’s cortical electrical activity. For this purpose, a 32-channel EEG was acquired (NeuroMax NMX 32) using a standard procedure [18]. FFT analysis measuring Power Spectral Density against the frequency using the Hamming window algorithm was subsequently used to extract an average peak frequency of 1.6 Hz over the frontal areas (Figure 1) and 0.9 Hz over the bilateral M1 (Figure 2).

Second treatment plan

After the routine screening for TMS compatibility and informed consent, the second plan was started. The new optimized treatment protocol consisted of three parts per single session, each complete session lasting for about 45 minutes:

(1) H4 coil: LFS with 1.6 Hz over bilateral PFC given at 120% of RMT of the right hand, delivering about 1,980 pulses, i.e., 62 trains of 1.6 Hz and 20 seconds duration with 5 seconds interval.

(2) H4 coil: LFS with 0.9 Hz over bilateral M1 given at 90% of RMT of the right hand, delivering about 1,200 pulses, i.e., 75 trains of 0.8 Hz and 20 seconds duration with 1 second interval.

(3) H7 coil: HFS with 10 Hz over the medial motor cortex given at 100% of RMT of the right foot, delivering about 800-1,200 pulses. This stimulation was assumed to affect the cortical control areas for bladder activity.

This composite treatment session was applied once daily for seven days, followed by four sessions given once every two days. At the end of this 15-day composite treatment, there was marked improvement in the gait and cognition in the patient. After the sixth treatment day, he was observed to be reading a book for about an hour, which he had not done for about five years. The MDS-UPDRS Part III, MMSE, and MoCA scores were 37/132, 22, and 24, respectively, indicating moderate motor impairment and mild cognitive impairment. The patient’s step length was 0.83 feet (10/12 ft), and the stride length was 1.66 feet (10/6 ft).

**Figure 3:**
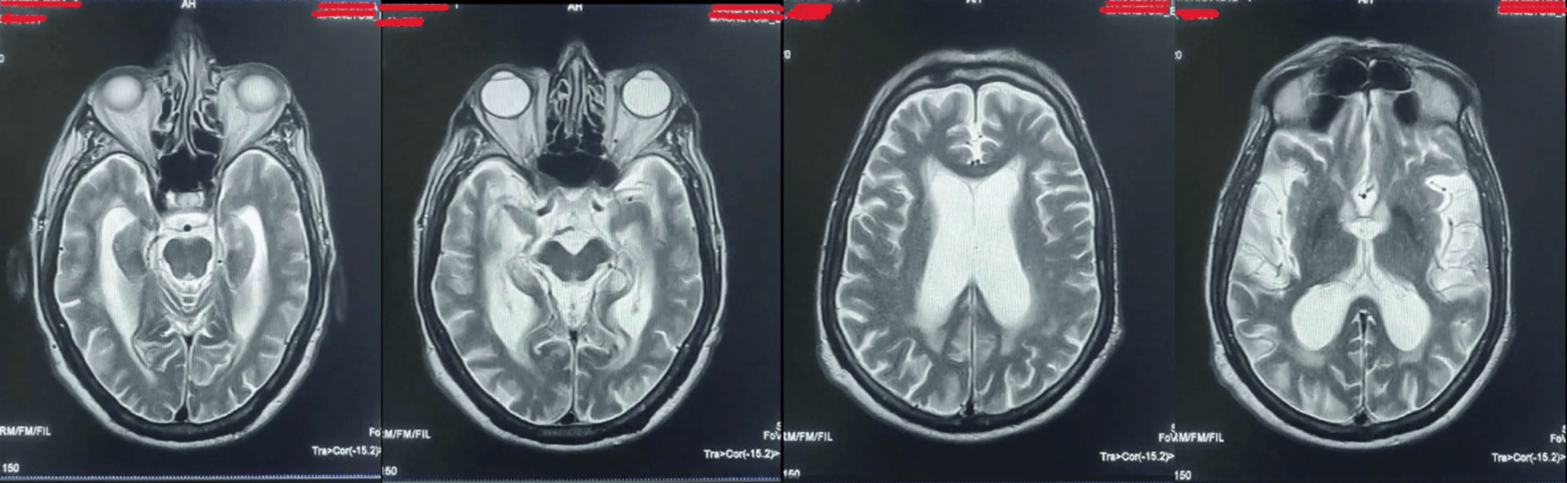
CT scan images showing normal lateral ventricles.

There was no change in the medication after the second treatment plan. In a review consultation that took place four days after the last treatment, improved urinary continence, marked cognitive improvement, and moderately improved gait were reported. Three months after the end of the treatment, the patient arrived having walked to the clinic unsupported. He was active, able to recollect the memories from his past as old as from 1970s. After about one year, nine more DTMS sessions, same as the second treatment plan, were given as maintenance, once daily. The MDS-UPDRSIII, MMSE, and MoCA scores at three-month and one-year visits were 35/132, 22, 23, and 30/132, 23, 25, respectively, indicating mild motor impairment and mild cognitive impairment. CTT was not performed during this period, and not since then. As of today, 13 months after the treatment, the patient is maintaining the improvement, with a perceived good quality of life. Along with routine medication, he is currently on Tablet Syndopa Plus (Levodopa 100 mg + Carbidopa 25 mg) along with all other routine medications. A recent CT scan conducted 18 months after the second DTMS treatment showed a normal ventricular system and brain parenchyma (Figure 3).

## Discussion

Non-invasive neuromodulation in the form of DTMS could possibly be a better treatment option in iNPH. The customized DTMS treatment was found to lower the burden of recurrent LPs, simultaneously giving a positive clinical outcome. The standard DTMS protocol for Parkinson’s disease was initially employed to target the gait and dyskinesia [16,17]. However, as the patient showed minimal improvement, the customized protocol became a viable approach, matching the treatment frequencies to the individualized frequencies measured in the patient. Measuring cortical activity by FFT is a routine practice applied to study the neuronal functions [19]. The same approach was applied to this patient and further extended to develop a personalized treatment protocol. The treatment regimen involved three stimulations: one over the bilateral motor cortex to reduce rigidity and improve gait, one over the medial areas of the motor cortex to target bladder incontinence, and one over the bilateral PFC to target cognitive symptoms. iNPH is characterized by inadequate absorption of CSF and consequent dilatation of the ventricular system to maintain an isobaric environment [1,2]. DTMS applied at very high stimulator output can affect the integrity of the blood-brain barrier via a glutamate-mediated mechanism [20]. A similar mechanism may have played a role in this patient by improving CSF absorption into the venous system. To our knowledge, no previously reported studies have applied DTMS in patients with iNPH.

## Conclusions

DTMS using H4 coil primarily, supported by H7 coil, has helped an old male patient with iNPH recover from his symptoms without the need for VPS surgery or recurrent LPs. The first set of maintenance sessions was given after about one year of the initial cycle. The use of this unique customized protocol in this patient with iNPH is promising but requires replication in further studies to establish a standardized DTMS treatment regimen.

## Figures and Tables

**Figure 4:**
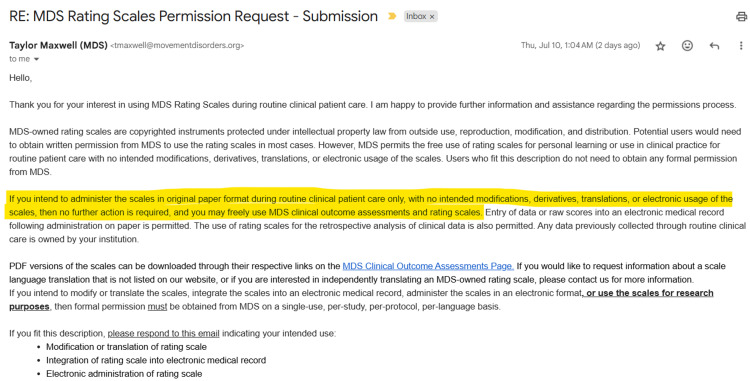
Figure 4: Screenshot of the permission e-mail to use MDS-UPDRS

**Figure 5:**
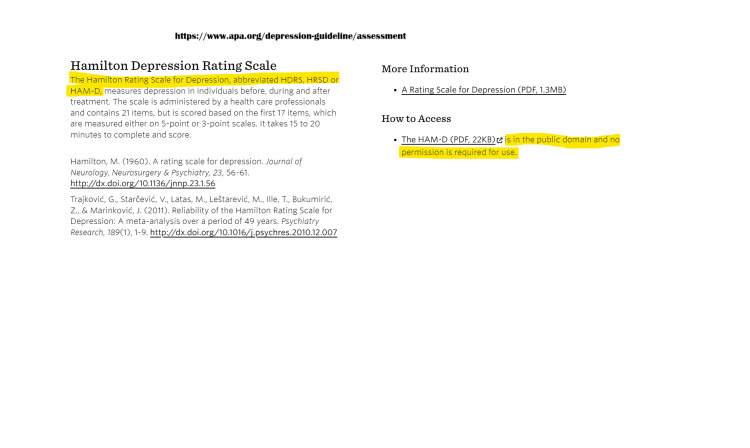
Figure 5: Screenshot highlighting that HDRS is in public domain

**Figure 6:**
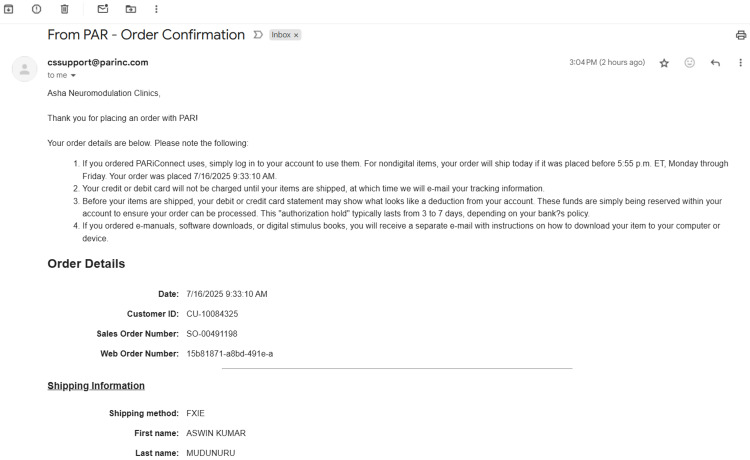
Page 1 of purchase order has been initiated with PAR for the usage of MMSE

**Figure 7:**
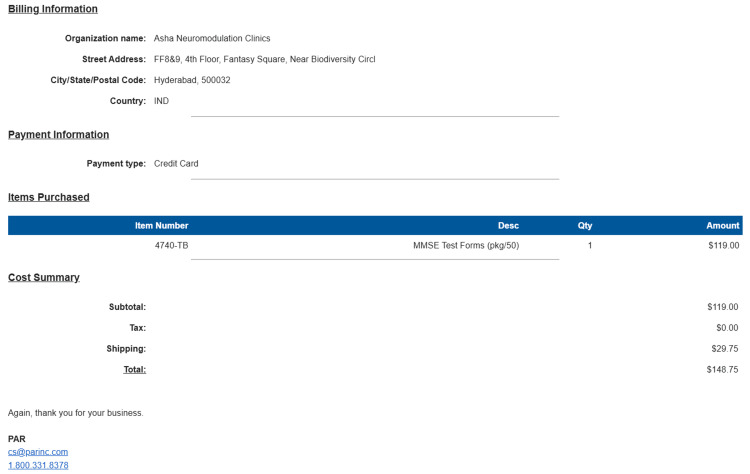
Page 2 of purchase order that has been initiated with PAR for the usage of MMSE

**Figure 8:**
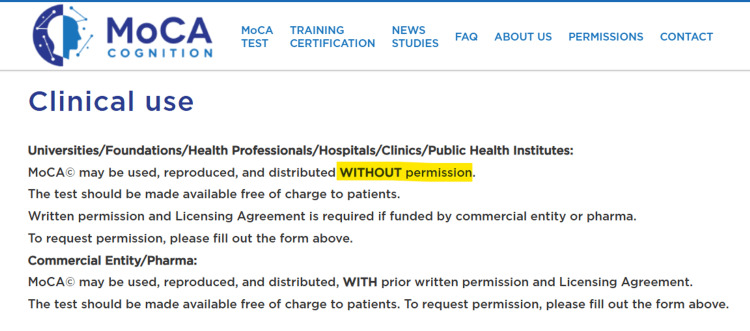
Screenshot showing that MoCA can be used without permission for clinical use

**Figure 9:**
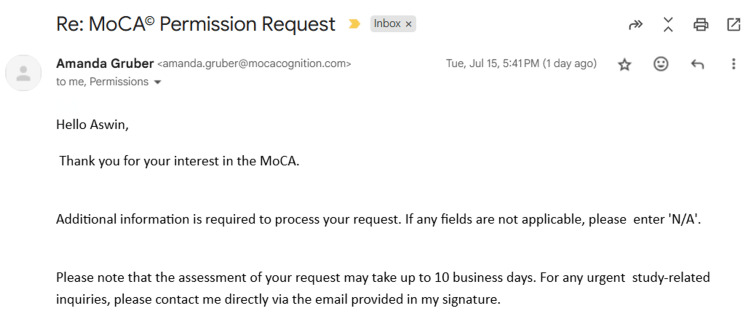
Screenshot showing e-mail communication with MoCA team for use in case report

## Appendices

Copyright and Permissions: Permission to use MDS-UPDRS has been acquired [Figure 4]. To the best of our knowledge, HDRS is available in the public domain and no permission is required [Figure 5]. As MMSE is copyright protected, we initiated the purchase of the MMSE forms and submission proofs are attached [Figures 7,8]. For MoCA, the email communication is attached [Figure 9,10].
